# 3-(4-Bromo­benzyl­idene)-1,5-dioxaspiro­[5.5]undecane-2,4-dione

**DOI:** 10.1107/S1600536811001516

**Published:** 2011-01-15

**Authors:** Wu-Lan Zeng

**Affiliations:** a MicroScale Science Institute, Department of Chemistry and Chemical Engineering, Weifang University, Weifang 261061, People’s Republic of China

## Abstract

The title mol­ecule, C_16_H_15_BrO_4_, was prepared by the reaction of (*R*)-2,4-dioxo-1,5-dioxaspiro­[5.5]undecane and 4-bromo­benzaldehyde with ethanol. The 1,3-dioxane ring exhibits a distorted boat and the fused cyclo­hexane ring exhibits a chair conformation.

## Related literature

For puckering parameters, see: Cremer & Pople (1975 ▶). For background information and related structures, see: Zeng & Jian (2009 ▶); Zeng *et al.* (2009 ▶).
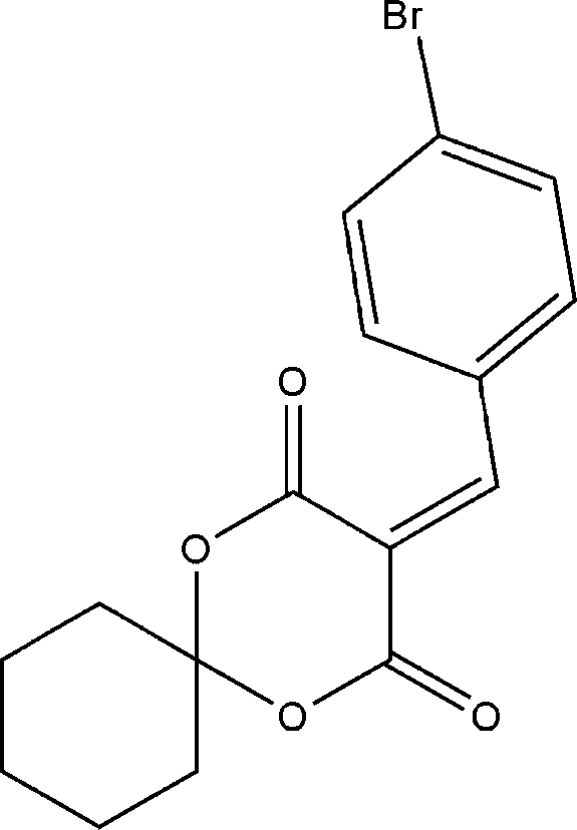


## Experimental

### 

#### Crystal data

C_16_H_15_BrO_4_
*M* *_r_* = 351.18
Orthorhombic, 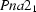

*a* = 6.6008 (13) Å
*b* = 16.784 (3) Å
*c* = 13.424 (3) Å
*V* = 1487.2 (5) Å^3^
*Z* = 4
Mo *K*α radiation
μ = 2.78 mm^−1^
*T* = 293 K
0.18 × 0.12 × 0.10 mm

#### Data collection

Bruker SMART CCD area-detector diffractometer
12420 measured reflections
3243 independent reflections
2042 reflections with *I* > 2σ(*I*)
*R*_int_ = 0.065

#### Refinement

*R*[*F* ^2^ > 2σ(*F* ^2^)] = 0.040
*wR*(*F* ^2^) = 0.124
*S* = 0.93
3243 reflections
191 parameters
2 restraints
H-atom parameters constrained
Δρ_max_ = 0.31 e Å^−3^
Δρ_min_ = −0.34 e Å^−3^
Absolute structure: Flack (1983 ▶), 1459 Friedel pairs
Flack parameter: −0.033 (13)

### 

Data collection: *SMART* (Bruker, 1997 ▶); cell refinement: *SAINT* (Bruker, 1997 ▶); data reduction: *SAINT*; program(s) used to solve structure: *SHELXS97* (Sheldrick, 2008 ▶); program(s) used to refine structure: *SHELXL97* (Sheldrick, 2008 ▶); molecular graphics: *SHELXTL* (Sheldrick, 2008 ▶); software used to prepare material for publication: *SHELXL97* and *PLATON* (Spek, 2009 ▶).

## Supplementary Material

Crystal structure: contains datablocks global, I. DOI: 10.1107/S1600536811001516/si2324sup1.cif

Structure factors: contains datablocks I. DOI: 10.1107/S1600536811001516/si2324Isup2.hkl

Additional supplementary materials:  crystallographic information; 3D view; checkCIF report

## Figures and Tables

**Fig. 1.**
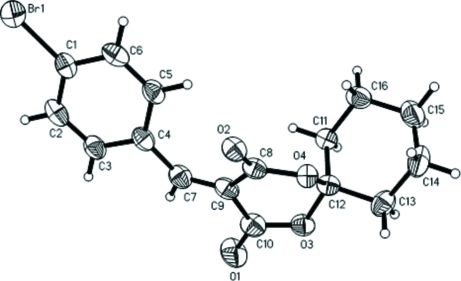
The molecular structure of (I), drawn with 30% probability ellipsoids and spheres of arbritrary size for the H atoms.

## supplementary crystallographic information

### Comment

We have recently reported the crystal structure of 3-(2-Furylmethylene)-1,5-dioxaspiro[5.5]undecane-2,4-dione (Zeng & Jian, 2009) and 3-[(5-Methylfuran-2-yl)methylene]-1,5-dioxaspiro- [5.5]undecane-2,4-dione (Zeng *et al.* 2009). As part of our ongoing studies on new spiro compounds with potentially higher bioactivity, the title compound, (I) (Fig. 1), has been synthesized and its structure is determined here. The crystal structure analysis confirms the title compound with atom C7 connected by the 1,3-dioxane ring via C4-C7 single bond [1.462 (7)Å] and the phenyl ring via C7═C9 double bond [1.328 (7)Å]. In (I) (Fig. 1), the 1,3-dioxane ring has a distorted boat conformation. The four atoms (O3/C10/C8/O4)are approximately planar with an rms deviation of 0.0279 Å. The max. deviation from the mean plane is 0.029 (1) Å, and atoms C9 and C12 deviate -0.245 (2)Å and -0.567 (2) Å, respectively. Thus, the cyclohexane ring exists in a chair comformation (Cremer & Pople, 1975) with the puckering parameters Q =0.560 (5)Å, θ =3.9 (5)°, φ = 312 (10)°.

### Experimental

The mixture of malonic acid (6.24 g, 0.06 mol) and acetic anhydride(9 ml) in strong sulfuric acid (0.25 ml) was stirred with water at 303 K. After dissolving, cyclohexanone (5.88 g, 0.06 mol) was added dropwise into the solution for 1 h. The reaction was allowed to proceed for 4 h. The mixture was cooled and filtered, and then an ethanol solution of 4-bromobenzaldehyde (11.04 g,0.06 mol) was added. The solution was then filtered and concentrated. Single crystals were obtained by evaporation of an ethanol solution of (I) at room temperature over a period of one week.

### Refinement

The H atoms were placed in calculated positions (C—H = 0.93–0.97 Å), and refined as riding with *U*_iso_(H) = 1.2*U*_eq_(C).

### Crystal data

**Table:**

C_16_H_15_BrO_4_	*F*(000) = 712
*M**_r_* = 351.18	*D*_x_ = 1.568 Mg m^−^^3^
Orthorhombic, *P**n**a*2_1_	Mo *K*α radiation, λ = 0.71073 Å
Hall symbol: P 2c -2n	Cell parameters from 2042 reflections
*a* = 6.6008 (13) Å	θ = 3.0–27.5°
*b* = 16.784 (3) Å	µ = 2.78 mm^−^^1^
*c* = 13.424 (3) Å	*T* = 293 K
*V* = 1487.2 (5) Å^3^	Block, yellow
*Z* = 4	0.18 × 0.12 × 0.10 mm

### Data collection

**Table:**

Bruker SMART CCD area-detector diffractometer	2042 reflections with *I* > 2σ(*I*)
Radiation source: fine-focus sealed tube	*R*_int_ = 0.065
graphite	θ_max_ = 27.5°, θ_min_ = 3.0°
φ and ω scans	*h* = −8→8
12420 measured reflections	*k* = −21→21
3243 independent reflections	*l* = −17→17

### Refinement

**Table:**

Refinement on *F*^2^	Hydrogen site location: inferred from neighbouring sites
Least-squares matrix: full	H-atom parameters constrained
*R*[*F*^2^ > 2σ(*F*^2^)] = 0.040	*w* = 1/[σ^2^(*F*_o_^2^) + (0.0603*P*)^2^] where *P* = (*F*_o_^2^ + 2*F*_c_^2^)/3
*wR*(*F*^2^) = 0.124	(Δ/σ)_max_ < 0.001
*S* = 0.93	Δρ_max_ = 0.31 e Å^−^^3^
3243 reflections	Δρ_min_ = −0.34 e Å^−^^3^
191 parameters	Extinction correction: *SHELXL97* (Sheldrick, 2008), Fc^*^=kFc[1+0.001xFc^2^λ^3^/sin(2θ)]^-1/4^
2 restraints	Extinction coefficient: 0.0088 (18)
Primary atom site location: structure-invariant direct methods	Absolute structure: Flack (1983), 1459 Friedel pairs
Secondary atom site location: difference Fourier map	Flack parameter: −0.033 (13)

### Special details

**Table:**

Geometry. All e.s.d.'s (except the e.s.d. in the dihedral angle between two l.s. planes) are estimated using the full covariance matrix. The cell e.s.d.'s are taken into account individually in the estimation of e.s.d.'s in distances, angles and torsion angles; correlations between e.s.d.'s in cell parameters are only used when they are defined by crystal symmetry. An approximate (isotropic) treatment of cell e.s.d.'s is used for estimating e.s.d.'s involving l.s. planes.
Refinement. Refinement of *F*^2^ against ALL reflections. The weighted *R*-factor *wR* and goodness of fit *S* are based on *F*^2^, conventional *R*-factors *R* are based on *F*, with *F* set to zero for negative *F*^2^. The threshold expression of *F*^2^ > σ(*F*^2^) is used only for calculating *R*-factors(gt) *etc*. and is not relevant to the choice of reflections for refinement. *R*-factors based on *F*^2^ are statistically about twice as large as those based on *F*, and *R*- factors based on ALL data will be even larger.

### Fractional atomic coordinates and isotropic or equivalent isotropic displacement parameters (Å^2^)

**Table:**

	*x*	*y*	*z*	*U*_iso_*/*U*_eq_	
Br1	−0.30705 (7)	0.16937 (3)	0.44191 (6)	0.0845 (2)	
O4	0.3239 (4)	0.18964 (18)	−0.1213 (2)	0.0495 (7)	
C8	0.2952 (5)	0.1895 (3)	−0.0213 (4)	0.0474 (9)	
C10	0.5373 (7)	0.0731 (3)	−0.0208 (4)	0.0683 (13)	
O3	0.5363 (4)	0.07733 (19)	−0.1204 (2)	0.0628 (8)	
O2	0.2192 (4)	0.24707 (17)	0.0163 (3)	0.0565 (7)	
C7	0.3358 (7)	0.0974 (3)	0.1252 (4)	0.0625 (11)	
H7A	0.4214	0.0577	0.1490	0.075*	
C4	0.1814 (6)	0.1207 (3)	0.1977 (4)	0.0562 (11)	
C9	0.3812 (7)	0.1202 (3)	0.0332 (4)	0.0574 (11)	
C12	0.3662 (6)	0.1150 (2)	−0.1684 (4)	0.0504 (10)	
C6	−0.1475 (7)	0.1696 (3)	0.2454 (5)	0.0654 (13)	
H6A	−0.2712	0.1920	0.2277	0.079*	
C5	−0.0046 (7)	0.1545 (2)	0.1731 (4)	0.0626 (11)	
H5A	−0.0327	0.1670	0.1071	0.075*	
C1	−0.1088 (7)	0.1517 (2)	0.3444 (3)	0.0569 (10)	
C13	0.4385 (7)	0.1343 (3)	−0.2731 (4)	0.0669 (13)	
H13A	0.5485	0.1725	−0.2697	0.080*	
H13B	0.4898	0.0862	−0.3043	0.080*	
C11	0.1829 (5)	0.0624 (2)	−0.1692 (3)	0.0540 (10)	
H11A	0.1366	0.0539	−0.1015	0.065*	
H11B	0.2182	0.0110	−0.1973	0.065*	
O1	0.6638 (6)	0.0322 (3)	0.0197 (3)	0.0997 (14)	
C3	0.2160 (7)	0.1015 (3)	0.2968 (4)	0.0635 (12)	
H3A	0.3378	0.0775	0.3143	0.076*	
C2	0.0755 (7)	0.1171 (3)	0.3698 (4)	0.0690 (12)	
H2A	0.1036	0.1046	0.4358	0.083*	
C16	0.0131 (6)	0.0998 (3)	−0.2304 (4)	0.0694 (13)	
H16A	−0.1028	0.0643	−0.2316	0.083*	
H16B	−0.0287	0.1495	−0.1997	0.083*	
C14	0.2691 (10)	0.1683 (3)	−0.3360 (5)	0.0853 (18)	
H14A	0.3159	0.1742	−0.4041	0.102*	
H14B	0.2334	0.2207	−0.3112	0.102*	
C15	0.0829 (9)	0.1154 (4)	−0.3347 (4)	0.0873 (17)	
H15A	−0.0252	0.1409	−0.3719	0.105*	
H15B	0.1140	0.0651	−0.3670	0.105*	

### Atomic displacement parameters (Å^2^)

**Table:**

	*U*^11^	*U*^22^	*U*^33^	*U*^12^	*U*^13^	*U*^23^
Br1	0.0827 (3)	0.0985 (4)	0.0723 (4)	0.0064 (2)	−0.0037 (3)	−0.0060 (3)
O4	0.0564 (16)	0.0396 (14)	0.0526 (15)	0.0002 (11)	−0.0015 (12)	0.0001 (14)
C8	0.046 (2)	0.043 (2)	0.0540 (17)	0.0020 (15)	−0.0062 (16)	−0.0023 (18)
C10	0.062 (2)	0.057 (3)	0.086 (4)	0.020 (2)	−0.009 (2)	−0.007 (2)
O3	0.0540 (16)	0.0653 (19)	0.069 (2)	0.0174 (14)	−0.0026 (15)	0.0004 (17)
O2	0.0606 (15)	0.0474 (16)	0.0615 (17)	0.0070 (12)	−0.0094 (14)	−0.0046 (14)
C7	0.069 (3)	0.055 (3)	0.064 (3)	0.0137 (19)	−0.015 (2)	0.001 (2)
C4	0.064 (3)	0.049 (2)	0.056 (3)	0.0003 (18)	−0.015 (2)	0.0050 (19)
C9	0.052 (2)	0.049 (2)	0.072 (3)	0.006 (2)	−0.014 (2)	0.001 (2)
C12	0.0482 (18)	0.040 (2)	0.063 (3)	0.0070 (16)	−0.0009 (19)	−0.005 (2)
C6	0.058 (2)	0.056 (3)	0.083 (4)	−0.0011 (18)	−0.019 (3)	0.010 (2)
C5	0.063 (2)	0.065 (3)	0.060 (3)	−0.0008 (19)	−0.013 (2)	0.016 (2)
C1	0.070 (2)	0.052 (2)	0.049 (2)	−0.0062 (18)	−0.011 (2)	0.0021 (19)
C13	0.075 (3)	0.059 (3)	0.067 (3)	0.010 (2)	0.019 (2)	0.007 (2)
C11	0.057 (2)	0.045 (2)	0.060 (3)	−0.0001 (15)	−0.0026 (18)	−0.004 (2)
O1	0.095 (2)	0.111 (3)	0.093 (3)	0.059 (2)	−0.022 (2)	0.002 (2)
C3	0.070 (3)	0.063 (3)	0.057 (3)	0.012 (2)	−0.017 (2)	0.002 (2)
C2	0.083 (3)	0.061 (3)	0.063 (3)	0.002 (2)	−0.029 (3)	−0.002 (2)
C16	0.058 (2)	0.074 (3)	0.076 (4)	0.005 (2)	−0.009 (2)	−0.016 (3)
C14	0.111 (4)	0.091 (4)	0.054 (3)	0.040 (3)	0.020 (3)	0.016 (3)
C15	0.091 (4)	0.112 (4)	0.059 (3)	0.031 (3)	−0.014 (3)	−0.017 (3)

### Geometric parameters (Å, °)

**Table:**

Br1—C1	1.875 (5)	C5—H5A	0.9300
O4—C8	1.355 (5)	C1—C2	1.391 (6)
O4—C12	1.431 (5)	C13—C14	1.513 (8)
C8—O2	1.201 (5)	C13—H13A	0.9700
C8—C9	1.487 (6)	C13—H13B	0.9700
C10—O1	1.210 (6)	C11—C16	1.525 (6)
C10—O3	1.338 (6)	C11—H11A	0.9700
C10—C9	1.487 (7)	C11—H11B	0.9700
O3—C12	1.441 (5)	C3—C2	1.374 (7)
C7—C9	1.328 (7)	C3—H3A	0.9300
C7—C4	1.462 (7)	C2—H2A	0.9300
C7—H7A	0.9300	C16—C15	1.497 (7)
C4—C3	1.387 (6)	C16—H16A	0.9700
C4—C5	1.392 (6)	C16—H16B	0.9700
C12—C11	1.498 (6)	C14—C15	1.517 (9)
C12—C13	1.520 (6)	C14—H14A	0.9700
C6—C5	1.377 (8)	C14—H14B	0.9700
C6—C1	1.385 (7)	C15—H15A	0.9700
C6—H6A	0.9300	C15—H15B	0.9700
			
C8—O4—C12	117.6 (4)	C12—C13—H13A	109.3
O2—C8—O4	118.3 (4)	C14—C13—H13B	109.3
O2—C8—C9	125.6 (5)	C12—C13—H13B	109.3
O4—C8—C9	115.8 (4)	H13A—C13—H13B	108.0
O1—C10—O3	118.8 (5)	C12—C11—C16	110.8 (4)
O1—C10—C9	124.1 (5)	C12—C11—H11A	109.5
O3—C10—C9	117.1 (4)	C16—C11—H11A	109.5
C10—O3—C12	118.3 (4)	C12—C11—H11B	109.5
C9—C7—C4	134.4 (4)	C16—C11—H11B	109.5
C9—C7—H7A	112.8	H11A—C11—H11B	108.1
C4—C7—H7A	112.8	C2—C3—C4	121.9 (4)
C3—C4—C5	117.8 (5)	C2—C3—H3A	119.0
C3—C4—C7	117.5 (4)	C4—C3—H3A	119.0
C5—C4—C7	124.5 (5)	C3—C2—C1	119.7 (5)
C7—C9—C10	117.2 (4)	C3—C2—H2A	120.2
C7—C9—C8	126.7 (4)	C1—C2—H2A	120.2
C10—C9—C8	116.1 (5)	C15—C16—C11	110.4 (4)
O4—C12—O3	109.8 (3)	C15—C16—H16A	109.6
O4—C12—C11	111.2 (3)	C11—C16—H16A	109.6
O3—C12—C11	112.0 (3)	C15—C16—H16B	109.6
O4—C12—C13	106.5 (3)	C11—C16—H16B	109.6
O3—C12—C13	105.2 (3)	H16A—C16—H16B	108.1
C11—C12—C13	111.8 (4)	C13—C14—C15	111.8 (4)
C5—C6—C1	120.6 (4)	C13—C14—H14A	109.3
C5—C6—H6A	119.7	C15—C14—H14A	109.3
C1—C6—H6A	119.7	C13—C14—H14B	109.3
C6—C5—C4	120.8 (5)	C15—C14—H14B	109.3
C6—C5—H5A	119.6	H14A—C14—H14B	107.9
C4—C5—H5A	119.6	C16—C15—C14	111.3 (4)
C6—C1—C2	119.1 (5)	C16—C15—H15A	109.4
C6—C1—Br1	120.4 (4)	C14—C15—H15A	109.4
C2—C1—Br1	120.4 (4)	C16—C15—H15B	109.4
C14—C13—C12	111.4 (4)	C14—C15—H15B	109.4
C14—C13—H13A	109.3	H15A—C15—H15B	108.0

### Hydrogen-bond geometry (Å, °)

**Table:**

*D*—H···*A*	*D*—H	H···*A*	*D*···*A*	*D*—H···*A*
C5—H5A···O2	0.93	2.45	3.004 (2)	117
C7—H7A···O1	0.93	2.40	2.812 (2)	106
